# Impact of Service-Learning on Physiotherapy Students: Exercise Programs for Patients with Heart Transplantation and Acute Coronary Syndrome—A Randomized Clinical Trial

**DOI:** 10.3390/jcm11154360

**Published:** 2022-07-27

**Authors:** Elena Marques-Sule, Oscar Chiva-Bartoll, Juan J. Carrasco, David Hernández-Guillén, Sofía Pérez-Alenda, Xavier Francisco-Garcés, Trinidad Sentandreu-Mañó, Jesús Blesa

**Affiliations:** 1 Physiotherapy in Motion, Multispeciality Research Group (PTinMOTION), Department of Physiotherapy, University of Valencia, 46010 Valencia, Spain; juan.j.carrasco@uv.es (J.J.C.); sofia.perez-alenda@uv.es (S.P.-A.); 2 Department of Physiotherapy, University of Valencia, Gascó Oliag 5, 46010 Valencia, Spain; david.hernandez@uv.es (D.H.-G.); trinidad.sentandreu@uv.es (T.S.-M.); 3 Department of Education and Specific Didactics, Faculty of Human and Social Sciences, University Jaume I. Avda. Sos Baynat, s/n, 12005 Castellon, Spain; ochiva@uji.es; 4 Intelligent Data Analysis Laboratory, University of Valencia, Av. Universitat, s/n, Burjassot, 46100 Valencia, Spain; 5 Faculty of Physical Activity and Sports Sciences, University of Valencia, 46010 Valencia, Spain; xafran2@alumni.uv.es; 6 Nutrition and Food Chemistry, Faculty of Pharmacy, University of Valencia, Avda. Vicent Andrés Estellés, s/n, Burjassot, 46100 Valencia, Spain; jesus.blesa@uv.es

**Keywords:** physiotherapy, community health services, service-learning, education, teaching-learning methodology

## Abstract

Introduction. Service-learning (SL) seems to be useful for healthcare students. This study aimed at comparing an SL program versus a traditional approach (TA) on moral sensitivity, ethical competences, knowledge, teaching quality, SL participation and performance, and satisfaction in physiotherapy students. Methods. Randomized clinical trial. A total of 32 physiotherapy students were allocated to an SL group (SLG, *n* = 16), or to a TA group (TAG, *n* = 16). Participants had to create an exercise program for patients with heart transplantation and acute coronary syndrome. The SLG performed the program with real patients, whilst the TAG performed a traditional collaborative approach. Moral sensitivity, ethical competences, knowledge and teaching quality were assessed pre- and post-intervention. SL participation and performance and satisfaction of the SLG were assessed post-intervention. Results. After the intervention, the SLG showed higher moral sensitivity, ethical competences, knowledge and reported better teaching quality than the TAG. The SL program was useful, addressed a real need, contributed to learning, helped to solve problems, facilitated better comprehension, increased motivation, and students would like to use SL in other subjects. Conclusions. The SL program had a positive impact on students, encouraging the implementation of similar SL interventions. SL may be considered a teaching–learning methodology of choice in physiotherapy students.

## 1. Introduction

Service-learning (SL) is an experiential education method whereby learning occurs as a result of activities carried out in the community and based on needs. An SL program encourages students to participate in real-world problem solving while fostering civic responsibility and recognizing that solving complex problems requires multiple perspectives [1]. This methodology is based on three basic principles: (a) experience must be directly related to the content of the academic course, (b) students must contribute in a positive way to the community, and (c) students must reflect on their experience [2]. The benefits of SL have been well documented in the literature. The participation of university students in SL projects reports improved levels of self-efficacy, identity and moral development [3,4]; deeper understanding of course content [1]; and civic responsibility [5]. There are several differences between SL and clinical placements. First, SL interventions are not included in the usual care provided in the healthcare system. Second, SL aims at improving the knowledge of students regarding academic subjects. Therefore, SL is not related to the application of the whole professional sphere, but to the concrete aspects of a subject. Third, SL presents pedagogical features that are not present in the clinical placement (e.g., the reflective thinking and interaction with other students) [2].

SL has been applied in Health Sciences effectively. Global health programs have been carried out in rehabilitation [6], treatment of obesity [7], community engineering and physiotherapy [8], hearing rehabilitation [9], caring for disabled people [10], gerontology and the elderly [11,12], and child weight control [13].

Specifically, in the field of physical therapy, SL has been found to be useful not only to improve the understanding and practical application of theories learned in the classroom, but also to improve the professional skills necessary to become an efficient clinician [14,15]. In addition, it offers students an enriched quality education by enhancing collaboration and fostering the integration of knowledge by applying it to real-life situations [16]. Nevertheless, there is no research analyzing SL effects in physiotherapy students designing exercise programs for heart transplantation (HT) and acute coronary syndrome (ACS).

This study aimed at analyzing the effects on physiotherapy students of participation in an SL program implemented in patients with HT and ACS. Specifically, such effects focus on the analysis of variables identified in the scientific literature as being related to learning especially enhanced by SL: moral sensitivity, ethical and gender competences, knowledge, teaching quality assessment SL participatory evaluation, service performance and satisfaction.

## 2. Materials and Methods

### 2.1. Study Design

A parallel-group assessor-blinded randomized clinical trial was performed. Thirty-two physiotherapy students were allocated to either an SL group (SLG, *n* = 16), or a traditional educational approach group (TAG, *n* = 16). Assessments were performed at baseline and at post-intervention. The trial was conducted following the CONSORT extension for pragmatic clinical trials [17]. The study protocol was approved by the Institutional Research Ethics Committee (H1542195894247). All procedures were according to the Declaration of Helsinki. All enrolled participants provided written informed consent prior to participation. The principles of voluntary participation, confidentiality and anonymity applied during the research process. The trial was registered at www.clinicaltrials.gov (10 February 2020) (registration number: NCT04261998).

### 2.2. Participants

A total of 32 students in the third year of the Degree of Physiotherapy (University of Valencia, Spain), aged 19–35 years, were recruited in March 2020. According to existing meta-analyses on SL research that analyze intervention programs [18,19], this is a medium-high sample size. Criteria for participants’ inclusion were to be studying the Physiotherapy Degree at the mentioned University and willingness to participate. Students with previous SL training were excluded. From a total of 40 students who fulfilled the inclusion criteria, 32 took part in the study. The main reason for exclusion was unwillingness to participate (*n* = 8).

### 2.3. Randomization and Masking

Randomization was conducted by an independent research assistant, not involved in the trial, who prepared a computer-generated random allocation sequence. From the randomization allocation, opaque, sequentially numbered envelopes were prepared containing the treatment group assigned for each participant. Group allocation was revealed to the study members once the participants completed all baseline procedures. The outcomes assessor was blinded to treatment group allocation. The blinded assessor collected all baseline and post-intervention measures and entered data.

### 2.4. Intervention

A physiotherapy teacher with over 10 years’ experience performed both teaching methodologies. The students had to develop a therapeutic exercise program for HT and ACS based on cardiac rehabilitation by searching scientific databases. Both groups performed a 20 h intervention over 12 weeks.

(a) SLG participated in an SL program with real patients. The intervention was as follows: 1) Explanation of the project and creating groups of four students and allocating a real patient to each group (1.5 h); (2) Bibliographic search, planning and structured script of contents (3 h); (3) Face-to-face session with patients (2 h): identifying the patient’s needs, from the perspective of comorbidities, health education and therapeutic exercise; (4) Regular meetings with the teacher (3 h), with the coordinators from two patient associations (Valencian Heart Institute, Valencian Heart Transplantation Association) and monitoring sessions with the patient (4 h). Meetings included a joint reflection process on the practice; (5) Autonomous work (individual and group) (5 h); (6) Oral presentation of the program, guided debate, and evaluation of the project (1.5 h).

(b) TAG performed a traditional intervention of collaborative work without real patients, as follows: (1) Explanation of the project and creation of groups with four students each (1.5 h); (2) Bibliographic search, planning and structured script of contents (3 h); (3) Regular meetings with the teacher (3 h); (4) Autonomous work carried out by the students (5 h); (5) Collaborative work carried out among the students of each group (6 h); (6) Oral presentation, discussion and resolution of doubts (1.5 h).

### 2.5. Outcome Measures

A baseline questionnaire was completed to gather students’ sociodemographic information (age, gender, marital status, access to the degree, other university degrees, employment status, hours worked a week). Assessments were conducted by a teacher trained in managing the evaluation tools.

### 2.6. Primary Outcome

(a) Moral sensitivity, measured with the Revised Moral Sensitivity Questionnaire (RMSQ) [20], that includes nine items using a 6-point Likert scale (1 = totally disagree, 6 = totally agree). A higher score reflects greater moral sensitivity. The questionnaire has been proven a valid instrument for assessing moral sensitivity.

### 2.7. Secondary Outcomes

(b) Cross-curricular ethical and gender competences, using the Higher Education, Transversal Skills and Gender Questionnaire [21]. The tool is composed of six items using a 7-point Likert scale (1 = totally disagree, 7 = totally agree) and has shown a high internal consistency. The higher the score, the better the ethical and gender competences.

(c) Knowledge acquisition, measured with an ad-hoc retention test. The test included 10 multiple-choice questions that assessed basic concepts of therapeutic exercise in HT and ACS that physiotherapy students are required to know (warm-up, exertion and cool-down; functional capacity parameters; rate of perceived exertion, etc.).

(d) Students’ teaching quality assessment, using the Teaching evaluation of the Centre for Quality of the University of Valencia. The survey carried out included different items evaluating the teaching methodologies, materials and general satisfaction on a Likert scale (1 = totally disagree, 5 = totally agree) in the undergraduate degree, through the University’s Virtual Office.

(e) SL participatory evaluation, using the Participatory Evaluation, SL and University Questionnaire [22], which comprises 25 items assessing aspects such as responsibility, dedication, attendance, punctuality, learning or implication in the SL program. Items are registered on a 5-point Likert format (1 = totally disagree, 5 = totally agree).

(f) Overall service performance, using the SL Questionnaire [23]. The tool comprises 16 items divided into 3 dimensions: service performance (6 items), competences achieved (2 items), and level of participation and satisfaction (8 items). It has shown an excellent internal consistency for service performance, competences achieved, level of participation and satisfaction.

(g) Satisfaction, using an ad-hoc questionnaire designed by the researchers of this study, comprising 22 items registered on a 5-point Likert format (1 = totally disagree, 5 = totally agree). The questions assessed SL methodology satisfaction, usefulness, influence on attendance to classes, skill improvement, teamwork, interest, overall satisfaction, etc.

Moral sensitivity, ethical and gender competences, knowledge and teaching quality assessment were measured at baseline and at post-intervention in both groups, whilst overall service performance, SL participatory evaluation, and satisfaction were assessed in the SL group at post-intervention.

### 2.8. Statistical Analysis

All analyses were carried out using IBM SPSS Statistics software (Version 22.0; IBM Corp., Armonk, NY, USA). Shapiro–Wilk test was used to verify the normality of the data. Descriptive data are shown as mean and standard deviation or frequencies, as appropriate. Age and gender from both groups were compared using the unpaired t-test and Chi-square (χ^2^), respectively. To evaluate the effect of the interventions on moral sensitivity, cross-curricular ethical and gender competences, and knowledge, a mixed two-factor ANOVA [time (pre and post) × group (SLG and TAG)] with repeated measures in the time factor was used. When the ANOVA indicated significant differences in the main effects, Bonferroni correction was applied to avoid type I error in the multiple comparisons. Effect size was interpreted as small (d = 0.2; ηp^2^ = 0.01), medium (d = 0.5; ηp^2^ = 0.06) and large (d > 0.8; ηp^2^ > 0.14). Statistical significance was set at *p* < 0.05.

## 3. Results

**Figure 1:**
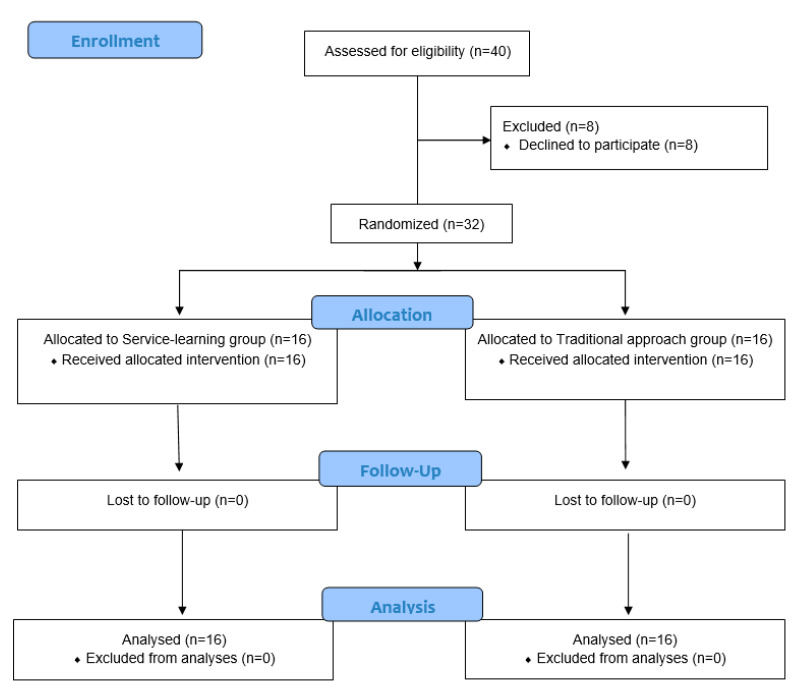
Flow diagram according to CONSORT statement for the report of randomized trial.

Forty subjects were eligible, and finally thirty-two were randomly assigned to SLG (*n* = 16) and TAG (*n* = 16) and completed the study (Figure 1). No significant differences (*p* > 0.05) were found between groups at baseline (Table 1).

### 3.1. Moral Sensitivity, Cross-Curricular Ethical and Gender Competences, and Knowledge

Significant interaction of the SL program in the time*group factor was observed in moral sensitivity [F(1, 36.0) = 16.9; *p* < 0.001; ηp^2^ = 0.36], cross-curricular ethical and gender competences [F(1, 30.3) = 18.6; *p* < 0.001; ηp^2^ = 0.38)] and knowledge [F(1, 27.6) = 10.3; *p* = 0.003; ηp^2^ = 0.26]. Mean (standard deviation) and post hoc analyses results of the intervention variables are shown in Table 2. After the intervention, the SLG presented a significantly higher score than the TAG in moral sensitivity, ethical and gender competences, as well as in knowledge. In all cases, the effect size was medium or high.

Regarding the within-group results, after the intervention, the SLG showed a significant improvement in the scores of all the questionnaires, with a large effect size. However, compared to pre-intervention, the TAG showed a significant improvement only in the knowledge test score, with a smaller effect size than the SLG.

### 3.2. Students’ Teaching Quality Assessment

The SLG rated higher certain items related to: structure of the activities performed (SLG: 4.5; TAG: 3.7), encouraging participation in the activities (SLG: 4.3; TAG: 3.7), while attendance to activities helped to study and understand the contents of the subject (SLG: 4.4; TAG: 3.7), and tutorships were useful (SLG: 4.7; TAG: 4.2). Moreover, the SLG showed higher general satisfaction (SLG: 4.5; TAG: 3.9), would better recommend the teacher to other students (SLG: 4.6; TAG: 4.0) and was more satisfied with the contents they had learnt (SLG: 4.5; TAG: 3.9).

### 3.3. SL Participatory Evaluation

The SLG reported scores > 4 out of 5 in 91.7% of the participatory evaluation items. The aspects best considered (score ≥ 4 out of 5) were: good relationship with colleagues (4.9 ± 0.5), punctuality (4.8 ± 0.4), acquisition of tools for the future development of the profession (4.7 ± 0.5), integration with peers (4.7 ± 0.5), attendance (4.6 ± 0.7), satisfaction with learning (4.6 ± 0.6), motivation (4.6 ± 0.5), good service attitude (4.6 ± 0.5), participation (4.6 ± 0.5) and responsibility (4.5 ± 0.5).

### 3.4. Overall Service Performance

Regarding the service performed, all projects assessed health promotion with a direct intervention by patients. The SL activities were considered as useful for inclusion in course contents (4.6 ± 0.6) and addressed a real need (4.5 ± 0.5). The usefulness of the SL activities (Table 3, dimension I) showed a mean score ≥ 4.3 out of 5 for all items.

With regard to the competences achieved, the SL project contributed to their learning of the subject (4.7 ± 0.7). Students rated 16 competences with a score ranging from 4 to 5 (Table 3, dimension II), highlighting the importance of solving problems and searching for and managing information.

Regarding the level of participation and satisfaction (Table 3, dimension III), the most frequent reasons that motivated students to participate were working with an organization, and preference for this type of project, with mean scores higher than 4.8 out of 5. Coordination between teachers and organization was highlighted.

### 3.5. Satisfaction

The students reported that SL led to a better comprehension of subject contents (4.8 ± 0.4), helped to promote greater interest in therapeutic exercise in heart disease (4.7 ± 0.5), and they would like to use SL in other subjects (4.7 ± 0.6) (Table 4).

## 4. Discussion

SL has turned out to be an innovative and effective teaching method for the improvement of subject learning in physiotherapy students, since it complements the learning provided through the academic curriculum and also the information and knowledge learnt through clinical placements. The specificity of the applied learning, the reflective thinking and the promotion of teamwork and social interaction are important to better understand the findings obtained in the present article. SLG presented higher scores than TAG in moral sensitivity, ethical and gender competences and knowledge, and reported a better teaching quality assessment. In addition, the SL program was useful, addressed a real need, contributed to the learning, helped to solve problems, promoted better comprehension, increased motivation based on working with an organization, and students would like to use SL in other subjects.

SLG significantly improved moral sensitivity when compared to TAG. This improvement is aligned with the own conceptual frameworks that relate SL and physiotherapy [24]. In addition, the results are consistent with those from quantitative studies that report improvements in the development of moral identity, in the ethical decision-making process and in the recognition of ethical questions [25,26], as well as recent qualitative research such as that by [27,28], who refer to improvements in variables such as altruism, compassion and caring, social responsibility and advocacy. Consistent with the existing literature, our study shows that participation in SL is effective in improving the moral development of physiotherapy students involved.

Regarding the cross-curricular ethical and gender competences, our findings are in line with previous studies in the field of physiotherapy carried out with a quantitative [29,30], qualitative [25,31], and mixed-methods approach [32,33]. The scientific literature shows that participation in SL programs generates improvements in transversal ethical competences, civic engagement, ethical practice, identification of misconceptions and bias, and reinforcement of and sensitivity to cultural issues. Our results therefore add to this general trend, reinforcing the idea that SL is an effective teaching method to develop transversal skills and promote the comprehensive training of physiotherapy students.

In terms of knowledge, there are previous studies that examine the specific acquisition of knowledge from a number of health-related SL programs. In a global way, some studies [34] report improvements in clinical reasoning skills when compared to a control group. Along the same lines, other studies [35] point to statistically significant improvements in cognitive skills and ability to work with diverse populations. In the field of physiotherapy, several studies report results comparable to ours, such as that by Noonan et al. [36], which reports specific learning about non-pharmacological methods to treat musculoskeletal pain, or by Nordon-Craft et al. [12] that shows improvements in the ability to interpret results of evaluations to determine type and severity of balance impairments, or by Gazsi et al. [37] that reveals a better understanding of the practical application of the theoretical content due to SL. All these results confirm that SL is a method that offers guarantees when it comes to acquiring specific curricular learning both in the field of health in general and in the field of physiotherapy in particular.

On the other hand, the positive results obtained in the students teaching quality assessment represent a question that has also been analyzed in previous applications of SL in the health field. Specifically, in line with our results, the study by Thomas et al. [38] referred to a service focused on geriatric techniques, the students suggested that the experience was worthwhile and constituted an effective design. Likewise, Flinn et al. [35] report SL program results focused on health promotion in a community clinic, highlighting the students’ favorable perception of the SL educational program. However, as a counterpoint to these evaluations, there are also results that underline the existence of issues that could be improved in terms of the quality of some programs. For example, according to the study by Cokely et al. [9], students reported that, although SL facilitated the acquisition of theoretical and applied learning, there were certain points that should be improved in the development of the educational program, highlighting the great workload, the slowness of the learning process and the enormous investment of time required. Therefore, it is important to note that, although SL is usually well valued by students, there are elements that can be improved in some of the experiences analyzed.

With regard to the SL participatory evaluation, one of the best valued items was the acquisition of tools for the future development of the profession. This outcome is in line with the findings of a number of research studies focused on improving competences, such as the ability to apply knowledge in practice and basic knowledge of the profession. For example, several studies report improvements in general professional skills and attitudes [31,34,39]; as well as specific improvements in practical skills [37], professional values [40,41], and personal and professional growth [27,42]. In short, the number of documents published recently in this line shows the solid perception of students on the acquisition of learning related to their professional practice through SL.

Regarding overall service performance, our results showed that the SL program was useful, addressed real needs, helped in solving problems, led to a better learning and comprehension of contents, and students showed interest in using SL in other subjects. These findings reinforce those obtained by previous studies in the field of physiotherapy. These findings are supported by several other studies [35,43], stressing the students’ satisfaction with the significant learning they achieved, as well as socio-emotional learnings, improving interpersonal abilities and the capacity to face up to stressful situations.

Finally, in relation to satisfaction, in line with recent studies [27,31,44], it should be noted that some of the best valued factors in our research were the development of teamwork skills, as well as the possibility of applying content in practice. Overall, the SL experience offers many advantages for physiotherapy students, generating a remarkable level of satisfaction.

### Strengths and Limitations

This is the first study that analyzed SL effects in physiotherapy students designing exercise programs for HT and ACS. The SL group was in contact with real patients, which required more than sophisticated theorizing, and also required getting involved in real practice to learn what it actually takes to be a physiotherapist. As limitations, the sample is from a single university, thus it seems difficult to extrapolate the results to other countries. Future studies could use mixed methods, comparing results for quantitative and qualitative content.

## 5. Conclusions

The service-learning program had a positive impact on students, encouraging the implementation of similar service-learning interventions. University educators might consider these results to address teaching strategies, which could help students to acquire competences, knowledge and moral sensitivity when working with real patients, aspects that they will use in their future clinical practice as physiotherapists. Service-learning may be considered as the teaching–learning methodology of choice for physiotherapy students.

## Figures and Tables

**Table 1:** Participants characteristics.

	SLG (*n* = 16)	TAG (*n* = 16)	
Age (years)	20.5 (1.3)	21.0 (2.8)	*p* = 0.52 [−1.1:2.1]
Gender *n* (M/F)	8/8	11/5	χ^2^ (1) = 1.2; *p* = 0.28
Marital status *n* (single/married)	16/0	16/0	N. A.
Type of University access *n* (university admission exam/higher level education cycle)	15/1	15/1	N. A.
Other university degrees *n* (yes/no)	1/15	1/15	N. A.
Employment *n* (unemployed/work < 10 h a week/work from 10 to 20 h a week)	12/2/2	16/0/0	χ^2^ (2) = 4.6; *p* = 0.10

Data are expressed as mean (standard deviation) or *n*. Abbreviations: M = male; F = female; N. A. = not applicable; SLG = service-learning group; TAG = traditional approach group.

**Table 2:** Effect of the intervention on moral sensitivity, cross-curricular ethical and gender competences, and knowledge, for the Service-Learning Group and the Traditional Approach Group.

		Pre	Post	Pre vs. Post *p* [95% CI]; Effect Size
Moral sensitivity	SLG	37.8 (2.9)	41.2 (4.5)	**<0.001 [−4.4:-2.3]**; 1.93
	TAG	37.9 (3.1)	38.3 (3.0)	0.47 [−1.4:0.7]; 0.34
	SLG vs. TAG *p* [95% CI]; Effect size	0.95 [−2.3:2.1]; 0.02	**0.038 [0.2:5.7]**; 0.77	
Cross-curricular ethical and gender competences	SLG	37.3 (1.4)	40.2 (2.1)	**<0.001 [−3.9:−2.0]**; 3.19
	TAG	38.0 (0.9)	38.2 (2.3)	0.68 [−1.1:0.7]; 0.19
	SLG vs. TAG *p*0 [95% CI]; Effect size	0.08 [−1.6:0.1]; 0.64	**0.017 [0.4:3.6]**; 0.90	
Knowledge	SLG	2.6 (2.0)	7.0 (1.6)	**<0.001 [−5.6:−3.5]**; 1.55
	TAG	2.1 (2.0)	3.9 (1.9)	**0.005 [−2.9:−0.6]**; 0.82
	SLG vs. TAG *p* [95% CI]; Effect size	0.49 [−1.0:2.0]; 0.25	**<0.001 [1.9:4.4]**; 1.78	

Data are expressed as mean (standard deviation). Effect size with Cohen’s d. Statistically significant values are shown in bold. Abbreviations: CI = Confidence Interval; SLG = service-learning group; TAG = traditional approach group.

**Table 3:** Results of the overall service-learning performed (Service-Learning Questionnaire) for the Service-Learning Group.

**I.** **SERVICE PERFORMED:**	
Please rate the extent to which the SL activities that you carry out have been useful to you in:	Mean (SD)
Understanding social needs	4.3 (0.5)
2. Working with an organization, association, etc.	4.3 (0.6)
3. Awakening interest in social problems	4.5 (0.6)
4. Encouraging me to take part in the life of the faculty, neighborhood or town	4.4 (0.6)
5. Being more motivated to study	4.6 (0.5)
6. Learning course contents	4.5 (0.5)
7. Analysing and reflecting on course contents	4.3 (0.5)
8. Being more responsible for my own actions	4.7 (0.5)
9. Developing some of the competences in the degree	4.4 (0.5)
10. Contributing to a better society	4.5 (0.6)
11. Establishing relationships between theory and practice	4.5 (0.5)
12. Getting to know the professional field related to my degree	4.6 (0.5)
13. Understanding some course contents in greater depth	4.3 (0.5)
14. Developing values	4.4 (0.5)
**II.** **COMPETENCES ACHIEVED:**	
Please indicate the degree to which your SL project has contributed to developing the following transversal competences:	Mean (SD)
Familiarizing yourself with and understanding ideas and concepts	4.6 (0.5)
2. Organizing and planning	4.6 (0.5)
3. Analysing and summarizing	4.6 (0.5)
4. Taking decisions	4.4 (0.5)
5. Solving problems	4.7 (0.5)
6. Getting to know information and communication technologies	3.5 (0.9)
7. Searching for and managing information	4.7 (0.5)
8. Communicating orally and in writing	4.6 (0.5)
9. Learning foreign languages	1.1 (0.3)
10. Expressing feelings	3.8 (0.6)
11. Team working	4.8 (0.4)
12. Thinking critically	4.8 (0.4)
13. Developing ethical commitment	4.1 (0.6)
14. Recognizing diversity and multiculturality	4.5 (0.5)
15. Negotiating	3.5 (0.7)
16. Adapting to new situations	3.6 (0.6)
17. Being creative and innovative	3.9 (0.7)
18. Working independently	4.1 (0.6)
19. Leading others	3.5 (0.6)
20. Showing initiative and entrepreneurial spirit	4.4 (0.6)
21. Being concerned with quality and improvement	4.1 (0.7)
22. Developing awareness of the social and environmental impact of actions	4.7 (0.5)
23. Designing and managing projects	4.8 (0.4)
24. Assessing the sustainability of proposals and actions	3.6 (0.8)
**III.** **LEVEL OF PARTICIPATION AND SATISFACTION:**	
Please evaluate to what extent each of these reasons motivated you to take part in this project:	Mean (SD)
Because I like this type of project	4.8 (0.4)
2. To work with an organization, association, etc.	4.8 (0.4)
3. To put course contents into practice	4.7 (0.5)
4. To help/work with others	4.5 (0.5)
5. To be a member of an organization, association, etc.	3.8 (0.7)
6. To contribute to a better society	3.9 (0.9)
Please rate your degree of satisfaction with each of the following factors:	Mean (SD)
6. Geographical distance	3.9 (0.8)
8. Involvement of the organization	3.8 (0.7)
9. Timetable of activities	4.1 (0.8)
10. Type of activities to carry out	4.2 (0.8)
11. Coordination between teachers and organization	4.4 (0.7)
12. People I work with	3.8 (0.7)
13. Teachers’ monitoring of work	4.2 (0.8)

Abbreviations: SD = standard deviation; SL = service-learning.

**Table 4:** Results of the satisfaction questionnaire for the Service-Learning Group.

Items of the Questionnaire	Mean (SD)
1. I properly value the SL methodology	4.6 (0.5)
2. This learning methodology has helped me to better understand the contents of the subject	4.8 (0.4)
3. I consider this type of methodology useful for my learning	4.6 (0.5)
4. I believe that SL has allowed me to achieve the objectives of the subject	4.1 (0.8)
5. SL stimulates class attendance	4.1 (1.0)
6. The methodology used has allowed me to acquire values that can improve me as a student and as a person (commitment, respect, tolerance)	4.3 (0.8)
7. The methodology used allows acquiring and perfecting generic skills and competences useful in other social areas (information and communication technologies, information processing, summary drafting)	3.8 (0.9)
8. I believe that the SL methodology is better than traditional methodologies based mainly on master classes	4.4 (0.7)
9. With this learning tool I feel I am the main protagonist of my training	4.3 (0.7)
10. The SL methodology has encouraged teamwork	4.7 (0.5)
11. I feel that I have learned to work as part of a team after this experience	4.1 (1.0)
12. I feel that this group work has improved my ability to interact with others	3.6 (0.9)
13. Working in a group has had positive results for me	4.2 (0.8)
14. I would have liked to work individually	1.4 (0.5)
15. I consider the preparation and presentation of a physiotherapy treatment project through SL is useful for my learning	4.5 (0.7)
16. I consider developing a physiotherapy treatment project through SL is useful for developing my professional competence as a future physiotherapist	4.4 (0.6)
17. I prefer traditional learning, (individual), without participating in a team or in class	1.1 (0.3)
18. My satisfaction with the methodology used in this subject is high	4.3 (0.7)
19. I would like to use SL Strategies in other subjects of the Degree	4.7 (0.6)
20. I consider that it has helped me to become more interested in Physiotherapy and therapeutic exercise in cardiac pathologies	4.7 (0.5)
21. I think it has helped me to become more interested in the subject of Cardiocirculatory Physiotherapy	4.6 (0.6)
22. My interest in the subject has increased as a result of SL	4.6 (0.6)

Abbreviations: SD = standard deviation; SL = service-learning.

## Data Availability

Not applicable.
